# *Solanumplastisexum*, an enigmatic new bush tomato from the Australian Monsoon Tropics exhibiting breeding system fluidity

**DOI:** 10.3897/phytokeys.124.33526

**Published:** 2019-06-18

**Authors:** Angela J. McDonnell, Heather B. Wetreich, Jason T. Cantley, Peter Jobson, Christopher T. Martine

**Affiliations:** 1 Department of Biology, Bucknell University, 1 Dent Drive, Lewisburg, PA, USA Bucknell University Lewisburg United States of America; 2 Department of Biology, San Francisco State University, 1600 Holloway Avenue, San Francisco, CA, USA San Francisco State University San Francisco United States of America; 3 Northern Territory Herbarium, Alice Springs, Department of Environment and Natural Resources, Alice Springs, Northern Territory, 0870, Australia Department of Environment and Natural Resources Alice Springs Australia

**Keywords:** New species, andromonoecy, Solanaceae, Leptostemonum

## Abstract

A bush tomato that has evaded classification by solanologists for decades has been identified and is described as a new species belonging to the Australian “*Solanumdioicum* group” of the Ord Victoria Plain biogeographic region in the monsoon tropics of the Northern Territory. Although now recognised to be andromonoecious, *S.plastisexum* Martine & McDonnell, **sp. nov.** exhibits multiple reproductive phenotypes, with solitary perfect flowers, a few staminate flowers or with cymes composed of a basal hermaphrodite and an extended rachis of several to many staminate flowers. When in fruit, the distal rachis may abcise and drop. A member of SolanumsubgenusLeptostemonum, *Solanumplastisexum* is allied to the *S.eburneum* Symon species group. Morphometric analyses presented here reveal that *S.plastisexum* differs statistically from all of its closest relatives including *S.eburneum*, *S.diversiflorum* F. Meull., *S.jobsonii* Martine, J.Cantley & L.M.Lacey, *S.succosum* A.R.Bean & Albr. and *S.watneyi* Martine & Frawley in both reproductive and vegetative characters. We present evidence supporting the recognition of *S.plastisexum* as a distinctive entity, a description of the species, representative photographs, a map showing the distribution of members of the *S.eburneum* species group and a key to the andromonoecious *Solanum* species of the Northern Territory of Australia. This new species is apparently labile in its reproductive expression, lending to its epithet, and is a model for the sort of sexual fluidity that is present throughout the plant kingdom.

## Introduction

As one of the most species-rich angiosperm genera (Frodin 2004), *Solanum* L. is also fast growing, with more than 90 species described within the last decade alone (e.g. Gouvêa et al. 2019; Gouvêa et al. 2018; Agra 2008; Stehmann and Moreira 2016). Members of the genus occupy all continents except Antarctica and inhabit diverse niches. The “spiny solanums,” SolanumsubgenusLeptostemonum (Dunal) Bitter, make up the largest lineage (ca. 450 spp.) within the genus and have been the subject of much recent work related to their evolution, diversity and natural history (e.g. Bohs 2005; Levin et al. 2006; Anderson et al. 2007; Stern et al. 2011; Särkinen et al. 2013; Vorontsova et al. 2013; Vorontsova and Knapp 2016; Knapp et al. 2017; Aubriot et al. 2016; Martine et al. 2019).

The spiny solanums appear to have arrived in Australia some time in the last 5–10 million years (Särkinen et al. 2013) and have continued to radiate throughout the continent with a large portion of the currently known species diversity occurring in the upper third of the continent known as the Australian Monsoon Tropics (AMT) (Bean 2004). While this radiation is reflected in diverse plant morphologies (e.g. foliage, armature, trichomes/indument, growth form, seasonal habit), the most interesting variety in form may be related to reproductive biology (Symon 1970; Symon 1979a; Symon 1979b; Symon, 1981; Anderson and Symon 1988; Anderson and Symon 1989; Martine and Anderson 2007). In particular, the spiny solanums of the AMT exhibit three primary breeding systems (breeding system used here *sensu*Neal and Anderson 2005): 1) hermaphroditic species with perfect flowers (i.e. *S.quadriloculatum* F.Muell. and approximately 10 other species), 2) cryptically dioecious species that bear functional pollen and functional gynoecia on separate individuals (i.e. *S.dioicum* W.Fitzg. and approximately 12 other species) and 3) andromonoecious species with inflorescences bearing one perfect flower at the base of each inflorescence and several to many staminate flowers above it (i.e. *S.chippendalei* Symon and about 13 other species). Our research team has recently been interested in the evolution of the andromonoecious taxa and, through field and population-level genomic study (in prep), has recognised a new species that is evolutionarily and morphologically distinctive.

The new taxon is the latest in a series of novelties from a set of taxa and forms within the “andromonoecious bush tomato” clade (Martine et al. 2006; Martine et al. 2009) recently described from the region (e.g. Bean and Albrecht 2008; Martine et al. 2016; Lacey et al. 2017) and part of a larger lineage of andromonoecious, hermaphrodite and functionally dioecious species in the “*S.dioicum* + *S.echinatum* Group” (*sensu*Martine et al. 2019). Across the southern margin of the AMT, where many of these taxa are distributed, mosaic habitats and corresponding environmental pressures, coupled with climatic fluctuations over the last two million years (Bowman et al. 2010), appear to have driven speciation within *Solanum* and other plant lineages of the region (Edwards et al. 2017; Edwards et al. 2018; Jobson et al. in prep; Martine et al. unpublished data).

**Figure 1.**
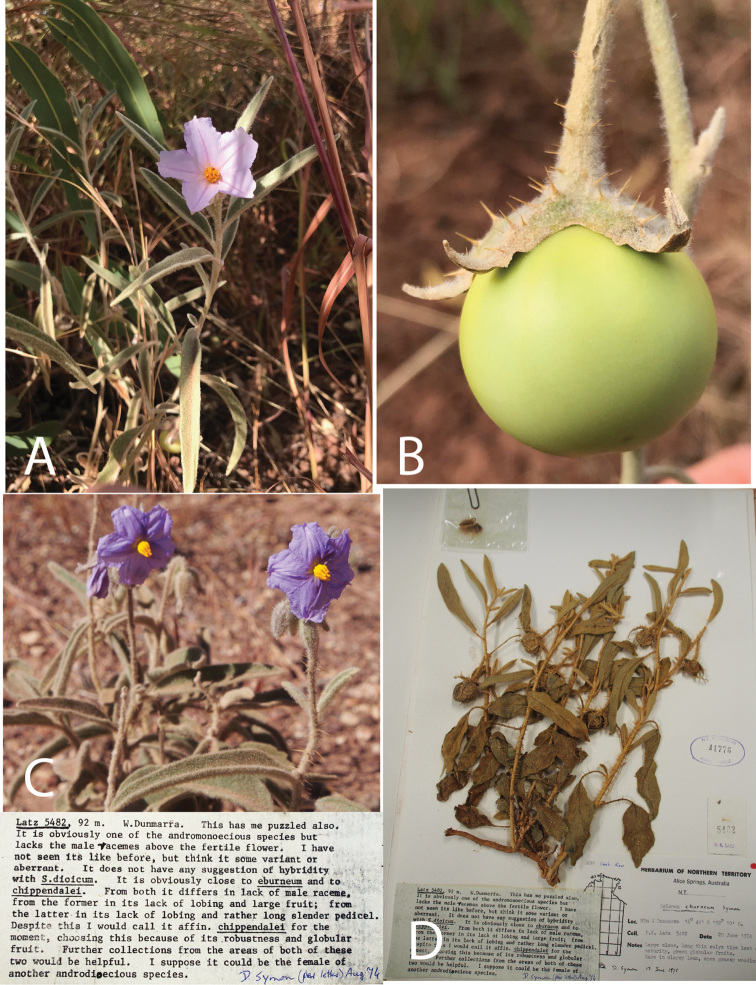
Morphology and the earliest-known herbarium specimen of *Solanumplastisexum*. **A** Flowering stem with a single staminate flower in 2016 **B** Mature fruit **C** Erect inflorescences bearing staminate flowers in 2018 and **D** Specimen collected by P. Latz in 1974, held at DNA and annotated by D. Symon with an annotation indicating his confusion about the reproductive morphology of the specimen (male rachis visible above fruit on far left).

In this paper, we describe *Solanumplastisexum* Martine & McDonnell, sp. nov., a new species restricted to a small area in the central region of the Northern Territory of Australia that has confounded field botanists since at least the early 1970s. The few historical collections made of this taxon were especially confusing to *Solanum* experts (Fig. 1) because plants may lack staminate flowers and/or the upper staminate rachis (typical for andromonoecious species) and it is often deciduous at fruit maturity. Recognition of this new species is supported by a suite of morphological characters including the lack of lobing on the leaves, a small apical leaf size and long-triangular calyx lobes on the staminate flowers. We include a morphometric comparison amongst closely-allied taxa, representative photographs, a distribution map and a key to the andromonoecious taxa of the Northern Territory along with the description of the new species.

## Materials and methods

Fieldwork in Northern Territory during 2016 facilitated collection of specimens with male flowers and tissue for population genomic study (in prep), which has revealed that this new entity is an independently evolving lineage (unpublished data). The same population was visited again in 2018 and facilitated the collection of specimens with complete andromonoecious inflorescences (including both male and hermaphrodite flowers) and mature fruits with viable seeds, as well as information about population size, extent and local ecology. Specimens were examined from BUPL, DNA and NT (herbarium acronyms follow Index Herbariorum; Thiers 2019). We consulted herbarium records with images via the Australasian Virtual Herbarium website (https://avh.chah.org.au/) and physically consulted specimens at the Northern Territory Herbarium in Darwin (DNA).

**Figure 2.**
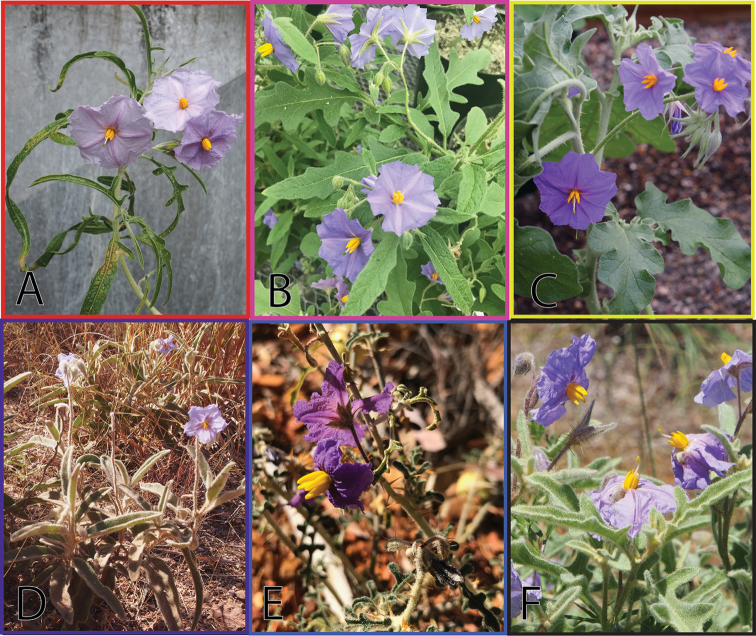
Closely related species of andromonoecious bush tomatoes included in this study. **A***Solanumjobsonii***B***S.watneyi***C***S.succosum***D***S.plastisexum***E***S.diversiflorum* and **F***S.eburneum*. Colours associated with each taxon also used in Figs 3, 4.

Field-collected seeds from two subpopulations were cultivated ex situ. First, seeds were soaked for 24 hours in 1000 ppm gibberellic acid solution in the dark at room temperature. Seeds were then sown in a growth chamber that was programmed to mimic an AMT climate and light regime at Bucknell University (Pennsylvania, USA) for approximately one month. Following successful growth, plants were cultivated in an IPM-managed greenhouse. Twenty-four vegetative and reproductive characters were measured from herbarium specimens and living plants. Characters were compared amongst six species that form a monophyletic group based on a recent phylogenetic and phylogenomic study (Martine et al. 2019; Martine et al. unpublished data): *S.diversiflorum* F.Meull., *S.eburneum* Symon, *S.jobsonii* Martine, J.Cantley & L.M.Lacey, *S.succosum* A.R.Bean & Albr., *S.watneyi* Martine & Frawley and this new species (Fig. 2). Some of the data for *S.eburneum*, *S.watneyi* and *S.jobsonii* have been published previously (Martine et al. 2016; Lacey et al. 2017). Those data were supplemented and measurements from the new species and *S.succosum* were newly collected as a part of this study. All characters were measured on mature plants and/or specimens collected from mature plants that included fully expanded apical and basal leaves; apical refers to expanded leaves near tips of growing stems while basal refers to expanded leaves on lower parts of stems.

Comparison of characters was conducted using JMP Pro 12 (SAS Institute, Inc., Cary, North Carolina, USA). Analyses included one-way ANOVA with Student’s t-test mean comparison at P < 0.05 and all the pairs by Tukey HSD to compare means and discern which species are different and in what way. A Connecting Letters Report was also generated to summarise mean values of each character across the six taxa included and to determine and assign significantly different sets when applicable. Multivariate morphometric analysis for all six taxa was also conducted using a principal components analysis (PCA) to place morphological variation in a spatial context.

## Results

ANOVA comparisons of each character along with Student’s t-tests and the Tukey HSD post-hoc comparisons reveal that species of this complex are, in large part, morphologically distinct (Table 1). Each comparison was significant and the Connecting Letters Reports reveal which sets exist for each character. Three statistically significant characters distinguish *S.plastisexum* from its closest relatives: depth of lobing on the margins of the basal leaves, surface area of the apical leaves and calyx lobe length on staminate flowers. *Solanumplastisexum* leaves are essentially unlobed, it has apical leaves that have a surface area of less than 7 cm^2^ and the calyx lobes on male flowers are greater than 1.5 cm long. Other traits that distinguish *S.plastisexum*, although not statistically significant for all comparisons, are plants that are relatively tall (70 cm) due to the erect nature of the stems, inflorescences and leaves and the essentially unlobed apical leaves.

**Figure 3.**
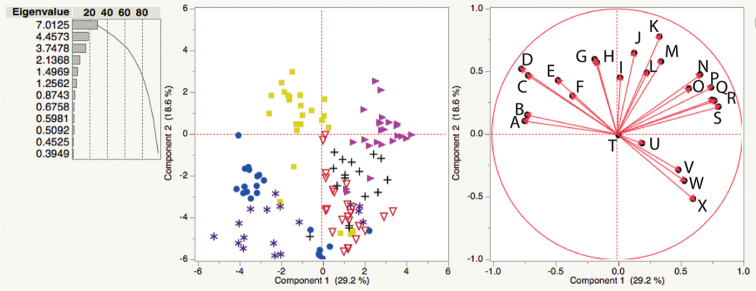
Principal components analysis score plot with eigenvalues and the contribution of each PC displayed (left) and loading plot (right) of characters and species in Table 1. Left, red triangles = *S.jobsonii*, blue circles = *S.diversiflorum*, black crosses = *S.eburneum*, pink triangles = *S.watneyi*, yellow squares = *S.succosum* and purple asterisks = *S.plastisexum*. Right, weighted characters labelled and indicated with red arrows. **A** Seed length **B** Fruit width **C** Number of seeds per fruit **D** Fruit length **E** Depth of lobing on apical leaves **F** Depth of lobing on basal leaves **G** Fruit wall width **H** Width of basal leaves **I** Surface area of basal leaves **J** Calyx lobe length, hermaphrodite flowers **K** Internode length **L** Width of apical leaves, **M** Surface area of apical leaves **N** Petiole length **O** Pedicel length in fruit **P** Corolla diameter, hermaphrodite flowers **Q** Length of basal leaves **R** Corolla diameter, male flowers **S** Length of apical leaves **T** Plant height **U** Stem prickle length **V** Trichome density, abaxial surface of apical leaves **W** Trichome density, adaxial surface of apical leaves **X** Calyx lobe length, male flowers. Colours associated with each taxon also used in Figs 2, 3.

The PCA score plot includes all measured characters and supports the relative distinctive nature of *S.plastisexum* when compared to the other taxa sampled. The analysis identified six eigenvalues above 1.0, which reveals that our dataset is roughly six-dimensional with principal components 1 and 2 contributing most of the variation amongst the points (47.8%). Figure 3 shows the score plot with each data point plotted along with the loading plot and shows which characters had the greatest weight in placing each of the points on the PCA.

**Table 1.** Vegetative and reproductive characters measured for species included in this study along with associated means (M), standard deviations (SD), sample sizes (n) and connecting letters reports (CL). Different letters in the CL for each character indicates distinctions between species; species not sharing the same letter in a row are significantly different for that character (p < 0.5). All measurements in cm, except for seeds per fruit (n), seed length (mm), fruit wall width (mm), surface areas (cm^2^) and trichome densities (per 0.5 cm^2^). The term apical refers to expanded leaves near tips of growing stems, while the term basal refers to expanded leaves on lower parts of the stems. Connecting letters values in bold text for *S.plastisexum* indicate characters that statistically differentiate the species from its closest relatives.

	* S. diversiflorum *				* S. eburneum *				* S. jobsonii *				* S. succosum *				* S. watneyi *				*S.* sp. nov.			
**Character**	**M**	**SD**	**n**	**CL**	**M**	**SD**	**n**	**CL**	**M**	**SD**	**n**	**CL**	**M**	**SD**	**n**	**CL**	**M**	**SD**	**n**	**CL**	**M**	**SD**	**n**	**CL**
Stem prickle length	0.22	0.09	30	D	0.4	0.1	16	A	0.29	0.12	54	BC	0.37	0.07	20	AB	0.26	0.1	24	CD	0.38	0.12	25	A
Internode length	2.23	0.91	30	B	2.16	0.67	16	B	1.48	0.51	54	C	3.6	0.73	25	A	4.01	0.98	24	A	1.48	0.43	25	C
Petiole length	1.03	0.53	30	D	2.68	0.8	16	B	0.82	0.39	54	D	1.78	0.44	25	C	3.36	0.78	24	A	0.79	0.32	25	D
Apical leaf length	2.66	0.77	25	C	11.32	1.83	16	A	5.39	1.33	25	B	5.13	1.09	25	B	12.39	2.51	24	A	4.97	0.89	25	B
Apical leaf width	1.61	0.41	25	B	1.41	0.41	16	BC	1.69	1.13	25	B	1.84	0.53	25	B	2.47	0.65	24	A	1.06	0.22	25	C
Basal leaf length	5.80	1.98	25	D	13.66	2.66	16	B	9.42	1.84	25	C	9.23	2.27	25	C	16.8	3.86	24	A	8.58	1.37	25	C
Basal leaf width	3.24	0.91	25	C	2.03	0.88	16	D	4.97	1.54	25	A	4.56	1.17	25	AB	3.97	1.16	24	BC	1.59	0.37	25	D
Trichome density, adaxial, apical leaves	81.6	48.28	5	B	419	125.27	5	A	121.0	61.42	5	B	345.0	43.36	5	A	138.33	20.26	3	B	482.2	79.82	5	A
Trichome density, abaxial, apical leaves	162.4	82.84	5	B	453.2	103.03	5	A	156.40	47.65	5	B	412.6	63.31	5	A	206.0	20.78	3	B	491.6	79.01	5	A
Depth of lobing, apical leaves	0.79	0.24	15	A	0.61	0.36	20	AB	0.9	0.62	15	A	0.91	0.4	25	A	0.27	0.36	10	BC	0.02	0.05	14	C
Depth of lobing, basal leaves	1.48	0.38	15	AB	1.31	0.67	12	B	2.01	0.92	15	A	1.22	0.49	18	B	0.87	0.59	6	B	0.02	0.06	14	**C**
Surface area, apical leaves	2.54	1.17	25	C	3.49	2.54	25	C	3.02	1.93	25	C	9.82	3.08	20	A	10.67	6.54	25	A	6.52	2.59	20	**B**
Surface area, basal leaves	9.99	4.12	25	B	7.71	1.84	7	B	19.86	7.68	25	A	23.91	9.74	20	A	16.83	7.51	4	AB	11.63	6.58	11	B
Corolla diameter, male flowers	2.24	0.45	16	D	3.5	0.31	15	B	3.02	0.44	23	C	3.11	0.34	25	BC	3.97	0.62	25	A	3.07	0.62	9	BC
Corolla diameter, hermaphrodite flowers	2.96	0.37	16	D	4.12	0.35	13	B	3.58	0.5	17	C	3.77	0.45	17	BC	4.69	0.58	22	A	3.54	0.33	4	BCD
Calyx lobe length, male flowers	0.35	0.09	15	E	0.69	0.06	3	D	1.16	0.14	10	C	0.17	0.02	25	F	1.13	0.14	7	B	1.63	0.03	3	**A**
Calyx lobe length, hermaphrodite flowers	0.35	0.09	6	D	0.9	0.15	5	C	1.65	0.14	14	C	2.41	0.33	19	A	1.78	0.52	4	B	1.72	0.05	4	B
Pedicel length, in fruit	2.7	0.18	13	C	3.64	0.93	14	AB	1.65	0.34	12	D	3.59	0.7	8	AB	4.16	0.72	17	A	2.94	0.47	9	BC
Fruit length	3.11	0.29	14	A	1.8	0.29	13	C	1.65	0.13	3	BC	2.88	0.26	21	A	2.15	0.34	29	B	2.0	0.37	17	BC
Fruit width	2.92	0.35	14	A	2.2	0.41	13	B	1.68	0.28	3	B	2.69	0.28	21	A	1.96	0.36	29	B	2.2	0.35	17	B
Seeds per fruit	433	–	1	A	78.69	36.67	13	C	101.67	58.6	3	C	262.81	48.26	20	B	53.11	28.45	28	C	70.08	49.87	13	C
Seed length	4.11	0.25	15	A	2.84	0.21	20	C	3.09	0.21	15	C	3.6	0.35	11	B	3.05	0.18	20	C	3.45	0.38	14	B
Fruit wall width	4.4	–	1	AB	3.1	–	1	AB	2.20	–	1	B	4.22	0.51	6	AB	5.5	–	1	A	3.35	0.49	2	AB
Plant height	33.8	5.35	3	B	43.62	10.86	16	B	34.08	7.3	6	B	69.2	27.03	5	A	45.85	6.91	24	B	77.33	2.52	3	A

### Taxonomic treatment

#### Solanum plastisexum

Taxon classificationPlantaeSolanalesSolanaceae

Martine & McDonnell sp. nov.

urn:lsid:ipni.org:names:77198273-1

Figs 1 , 2D , 5

##### Diagnosis.

Like *Solanumeburneum*, *Solanumwatneyi* and *Solanumsuccosum*, but differing by having elliptic, unlobed (or rarely very shallowly lobed) leaves, small apical leaves, long calyx lobes on the staminate flowers and fully erect staminate inflorescence branches.

##### Type.

AUSTRALIA. Northern Territory: ~42 km E of Top Springs, on and around the Buchanan Highway, 16°42.274'S, 132°07.446'E, elev. 286 m, 23 May 2016 (fl, fr), *C.T. Martine 4258, J.T. Cantley, L.M. Lacey, & P.C. Jobson* (holotype: DNA; isotypes to be distributed to BM, BUPL, MEL, NY, PERTH)

##### Description.

Erect perennial herb 50–80 cm tall. Stems slender, woody at base, upright even when weighted by fruits; single stemmed, with some lateral branching on mature stems. Foliage and stems grey to grey-green, becoming slightly more yellow-green with age; indumentum of stems, leaves and inflorescences composed of stellate trichomes with the stalk, these short, appressed and very dense throughout (of Type 1 *sensu*Bean 2004 and Seithe 1979); stalk 0.05–0.1 mm long, with 4–6 rays 0.2–0.4 mm long, the midpoint elongate, to 0.4 mm long. Prickles scattered throughout, tan, straight, slightly widened at base, fine, 1–2 mm long. Sympodial units difoliate, the leaves solitary or geminate. Mature leaves 3–12 cm × 0.7–2.4 cm, lanceolate (elliptic), with 3–7 pairs of primary veins, with few prickles along base of abaxial midvein; both sides closely and very densely stellate-pubescent; base tapering; margins entire, occasionally sparsely shallowly lobed; apex acute; petiole 0.3–1.8 cm long with 0–4 prickles along base of adaxial midvein. Inflorescence a supra-axillary cyme 1–15 cm long, complete inflorescence consisting of a basal hermaphrodite flower and a distal group of 2–many staminate flowers with 2–3 staminate flowers typically open at the same time; peduncle typically 1.0–10.0 mm long. Flowers 5-merous, heterostylous and the plants andromonoecious. Hermaphrodite flower ca. 1.5–3 cm below the oldest staminate flower, opening first; pedicel ca. 1 cm long at anthesis, elongating in fruit, armed with 20–50 prickles, each 1–3 mm long; calyx lobes 16–18 mm long, fused for first 2–3 mm, some occasionally fused along most of their length with sepals arranged 2+2+1, armed with 40 -100 long, straight prickles and dense stellate trichomes; corolla 3.1–4.0 cm in diameter, lavender to medium purple, rotate, glabrous; stamens equal; filaments ca. 1.0 mm long; anthers 5 mm long, oblong to lanceolate, poricidal, in a tight anther cone; ovary glabrous, ca. 1.5 mm diameter at anthesis; style (including capitate stigma) dimorphic, 2.5–5 mm or 7–10.5 mm long, curved. Staminate flowers with pedicels 4–7 mm long, unarmed; calyx lobes 16–17 mm long, fused at the base, occasionally 2+2+1 as in hermaphroditic flowers, prickles absent; corolla 1.7–3.5 cm in diameter, lavender to medium purple, rotate; stamens of same proportions as in hermaphrodite flowers; ovary, style and stigma vestigial and diminutive; rachis bearing staminate flowers often deciduous in fruit. Fruit a globose berry 1.4–2.7 cm long, 1.7–2.8 cm in diameter, light green with darker green striations when young, maturing to creamy yellow; flesh firm; locules 2, with little liquid; fruit wall ca. 3–4 mm thick; fruits retained on plant after maturation; fruiting pedicels 2.4–4.1 cm long; fruiting calyx covering 1/4 to 1/3 of developed fruit, the lobes narrowly deltoid, long-acuminate, tips acute, turning brown and weakly reflexing at maturity, very densely short stellate-pubescent and armed with sharp spines 2–5 mm long, these single or paired along the calyx sutures. Seeds up to ~150 per fruit, 3.0–4.5 mm long, dark brown to black, flat, reniform, the surfaces finely reticulate.

##### Distribution and ecology.

**Figure 4.**
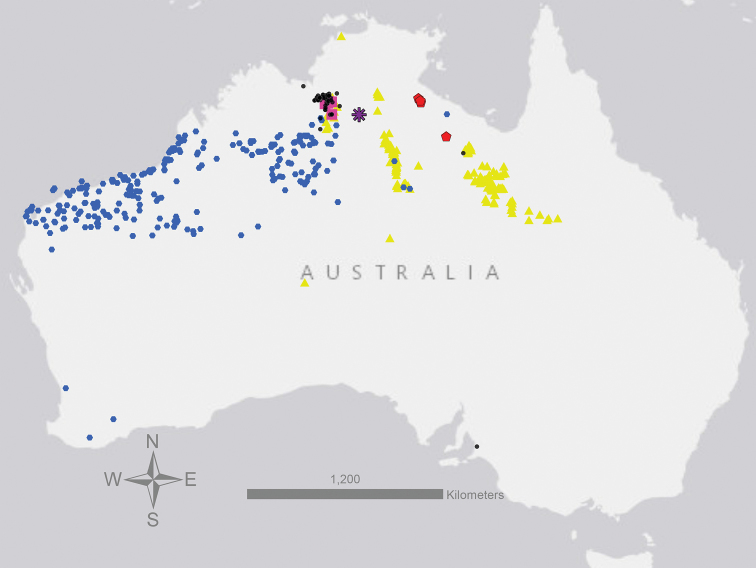
Map showing geographic distribution of all taxa compared in this study. red points = *S.jobsonii*, blue points = *S.diversiflorum*, black points = *S.eburneum*, pink points = *S.watneyi*, yellow points = *S.succosum* and purple asterisk = *S.plastisexum*. All points are based on specimens databased in the Australasian Virtual Herbarium (https://avh.chah.org.au/) and specimens held at BUPL.

*Solanumplastisexum* is currently known from a restricted range on and around the Buchanan Highway in the sub-arid, monsoon-influenced zone of the northern region of the Australian Northern Territory (Figs 4, 6). The species is locally abundant in two to three sites along and off of the road in *Corymbiadichromophloia* (F.Muell.) K.D.Hill & L.A.S.Johnson and *Petalostigmapubescens* Domin savanna woodland alongside *Aristida* L., *Heteropogoncontortus* (L.) P.Beauv. ex Roem. & Schult. and other herbaceous and shrubby associates. The distribution of plants along annually-graded roadsides suggests that the species is, like many of its congeners, disturbance-adapted.

Nothing is known about the biotic interactions local fauna have with this species, although the floral morphology suggests the typical *Solanum* buzz pollination syndrome (Anderson and Symon 1988). Plants encountered in 2018 bore many mature fruits not taken nor eaten by frugivores, but the exposed, fleshy berries may indicate biotic seed dispersal via ingestion (Martine et al. 2019).

##### Phenology.

The handful of collections that have been made of *S.plastisexum* that include flowers are all from the end of the wet season through the early months of the dry season, from January to June. Mature fruiting specimens have been collected in June.

##### Etymology.

The name is based on the Latin “plastus” (“deceptive,” but derived from the Greek “plastikos/plasticos/plasticus” for “able to be molded, changeable”) and the Latin “sexus” for sex. We suggest the use of Dungowan Bush Tomato for the common name of this species, which refers to the cattle station on which the majority of the collections have been made.

##### Preliminary assessment of conservation status.

*Solanumplastisexum* is known from only two to three extant populations, each consisting of a few dozen individuals (with some likelihood of clonality) and two historical (pre-2000) collections (Fig. 1D). The currently-known distribution of the species is not under conservation protection, but one of the populations appears to have been stable since at least the 1970s. When evaluated using the IUCN Red List Categories and Criteria for extinction risk (IUCN 2012), *S.plastisexum* falls into the Vulnerable (VU) category under Criterion B (B1ab(iii)+2ab(iii)). The VU designation is the lowest of three threatened categories, but indicates the taxon still faces a high risk of extinction in the wild. It has an Area of Occupancy that is likely much less than < 20 km^2^ and an Extent of Occurrence < 5 km^2^, as calculated using the geocat.kew.org online tool. There are fewer than 10 known locations that are possibly fragmented and a decline in overall habitat quality is likely, given population localities along a road.

##### Specimens examined.

**AUSTRALIA. Northern Territory**: 92 km W. of Dunmarra, 16°44'S, 132°10'E, 20 June 1974 (fr), *P.K. Latz 5482* (DNA [DNA A0041776]); Dungowan Station, 16°44'S, 132°17'E, 10 January 1978 (fl), *T.S. Henshall 1914* (DNA [DNA A0054101]); Buchanan Highway, 46.1 km E. of Top Springs, 16°43.140'S, 132°09.511'E, 22 May 2016 (fl), *C.T. Martine, J.T. Cantley, L.M. Lacey & P. Jobson 4260* (DNA, BUPL); Buchanan Highway, 42.1 km E. of Top Springs, 16°42.274'S, 132°07.446'E, 30 May 2018 (fl, fr), *C.T. Martine, A.J. McDonnell, J.T. Cantley, & P. Jobson 4743* (NT, DNA, BUPL); Buchanan Highway, 46.1 km E. of Top Springs, 16°43.140'S, 132°09.511'E, 30 May 2018 (fl, fr), *C.T. Martine, A.J. McDonnell, J.T. Cantley, & P. Jobson 4745* (DNA, BUPL).

**Figure 5.**
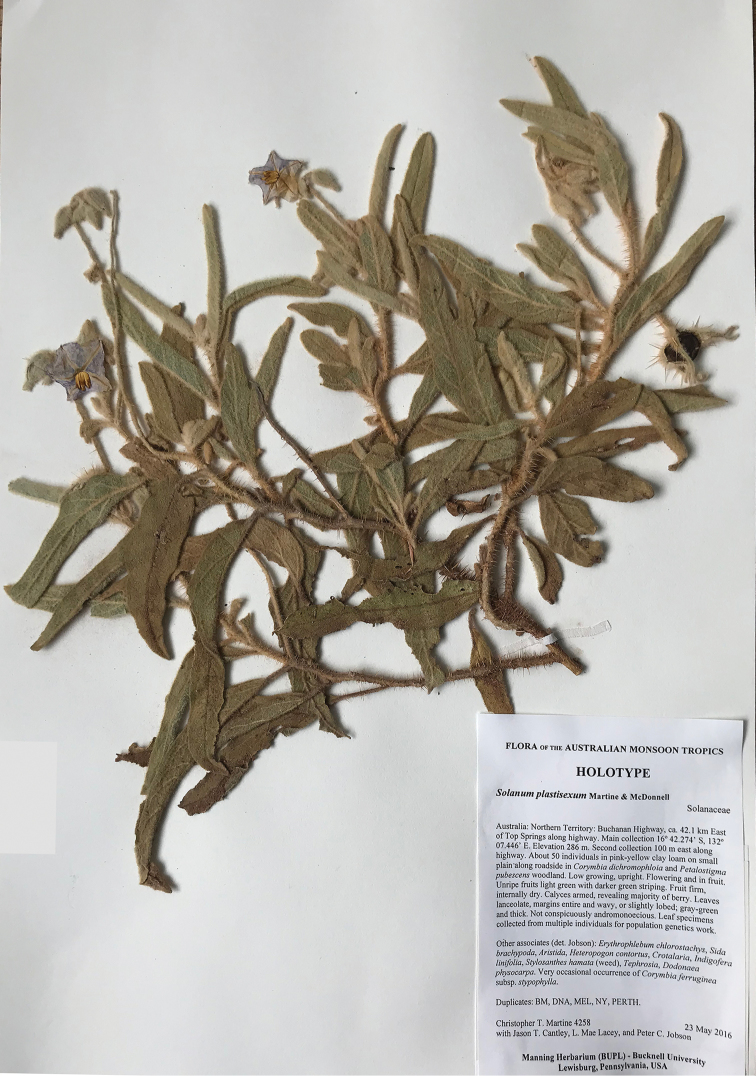
Scan of holotype of *Solanumplastisexum*, held at DNA.

**Figure 6.**
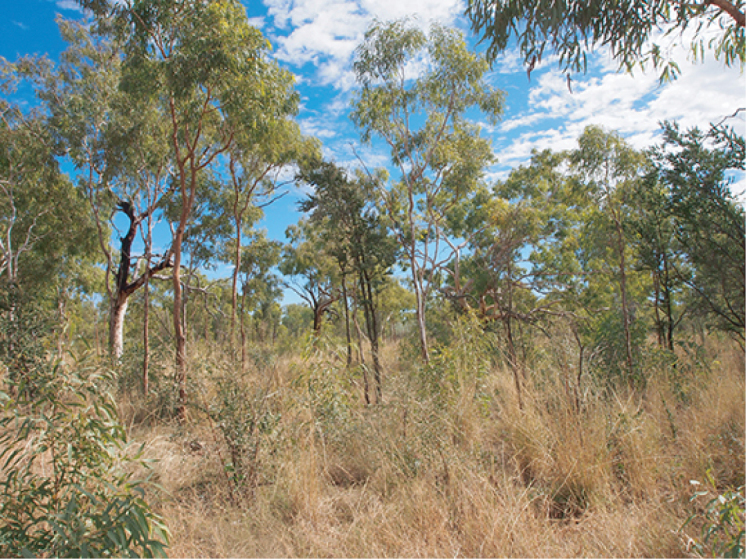
Typical habitat of *Solanumplastisexum*, taken from the Buchanan Highway.

### Field key to andromonoecious *Solanum* species of Northern Territory, Australia

Couplets 3 and 4 adapted from Bean and Albrecht (2008).

**Table:** 

1	Mature plants typically with stems 1 metre or more in height; plants possessing taproots, foliage deep green to yellow-green; occurring in the Top End region	**2**
–	Mature plants typically with stems 1 metre or less in height (or rarely ≥ 1 m); plants rhizomatous; foliage grey to blue green to deep green; primarily occurring south of Mataranka	**5**
2	Foliage yellow-green to rusty-green; leaves with several shallow or deep rounded lobes; plants perennial; typically along riverbanks around southern Gulf of Carpentaria	***S.melanospermum* F.Muell.**
–	Foliage bright or deep green; leaves ovate to oval with none or few pointed lobes; plants biennial; mostly restricted to northern/western escarpments of the Arnhem Plateau (and Wessel Islands)	**3**
3	Leaves sessile; stellate hairs on upper leaf surface with lateral rays more or less porrect (held horizontally)	***S.apodophyllum*** A.R.Bean
–	Leaves petiolate; stellate hairs on upper leaf surface mostly with ascending lateral rays	**4**
4	Fruiting calyx with 2300–2700 prickles; male flowers with pedicels 3–11 mm long	***S.ultraspinosum* A.R.Bean**
–	Fruiting calyx with 190–310 prickles; male flowers with pedicels 11–16 mm long	***S.clarkiae* Symon**
5	Erect herbs or shrubs (though branches may become lax and the plants sprawl slightly in fruiting stage)	**6**
–	Compact to weakly erect or sprawling herbs or shrubs	**9**
6	Plants greater than 0.5 metre in height at maturity	***S.succosum* A.R.Bean & Albr.**
–	Plants 0.5 metre or less in height at maturity	**7**
7	Leaf blades lanceolate; lobes, if any, with sinuses less than 0.2 cm in depth	***S.plastisexum* Martine & McDonnell**
–	Leaf blades elliptic, ovate or rarely lanceolate; lobes frequently with sinuses ≥ 0.5 cm in depth	**8**
8	Leaves dissected; leaf blades ovate to oblong, 2–4 cm long, sparsely pubescent	***S.diversiflorum* F. Muell.**
–	Leaves deeply lobed; leaf blades ovate to elliptic, 2.5–8 cm long, densely pubescent	***S.eburneum* Symon**
9	Leaves deep green; leaf blades linear, dissected, lanceolate or elliptic	**10**
–	Leaves grey-green; leaf blades ovate	**11**
10	Leaf blades linear to dissected with narrow lobes or ovate to elliptic and lobed; berries globose; restricted to eastern Northern Territory, Limmen National Park region	***S.jobsonii* Martine, J.Cantley, & L.M.Lacey**
–	Leaf blades lanceolate to elliptic and unlobed to shallowly lobed; berries ovoid; restricted to western Northern Territory, Bullita Homestead, Judbarra National Park and vicinity	***S.watneyi* Martine & Frawley**
11	Leaf margins shallowly lobed to entire, sinuate; fruit a dry berry	***S.chippendalei* Symon**
–	Leaf margins shallowly to deeply lobed, crenate to irregularly parted; fruit a juicy berry	**12**
12	Plants compact, typically much less than 0.5 m tall; restricted to north-western Northern Territory, west of Timber Creek, in the East Baines River corridor	***S.eburneum* Symon**
–	Plants weakly erect to sprawling, typically reaching 1 m tall; widespread in Northern Territory and western Queensland	***S.succosum* A.R. Bean & Albr.**

## Discussion

For at least five decades, the species described here has evaded easy classification by field botanists. The earliest known collections by Latz (*Latz 5482*, DNA) and Henshall (*Henshall 1914*, DNA) in the 1970s were each identified initially as S.aff.eburneum, with *Solanum* expert David Symon also suggesting S.aff.chippendalei for the former in an annotation (Fig. 1). Some of the confusion surrounding this taxon relates to the botanists’ inability to clearly identify its breeding system due to the species’ non-conformity to any one floral form and/or inflorescence type. Any given floral unit encountered in nature might consist of a fully andromonoecious inflorescence (basal bisexual flower with several distal staminate flowers), a solitary bisexual flower, a solitary short-styled (possibly functionally staminate) flower or an extended rachis of staminate flowers – with observers left to wonder whether any individual plant exhibits one of the three breeding systems found amongst its closest relatives; andromonoecy, hermaphroditism or functional dioecy. Labile sex expression has been observed in other *Solanum* lineages (e.g. Diggle 1991, 1994; Miller and Diggle 2003), but rarely to the same degree – and no other species amongst the ca. 30 taxa in the “*S.dioicum* + *S.echinatum* Group” (*sensu*Martine et al. 2019) shows this same plasticity relative to reproductive expression.

Given this apparent ability to exhibit elements of all three possible breeding systems, we have chosen the name *S.plastisexum*. This name is not just a reflection of the diversity of sexual forms seen in this species, but is also a recognition that this species could prove to be a model for the sort of sexual fluidity that is present throughout the plant kingdom – where just about any sort of reproductive form one can imagine (within the constraints of genetics and development) is present (Darwin 1877).

*Solanumplastisexum* is a new species that serves as an example of for the diversity of sexual/reproductive form that has been increasingly recognised amongst plants – it is also evidence that attempts to recognise a “normative” sexual condition amongst the planet’s living creatures is problematic. When considering the scope of life on Earth, the notion of a constant sexual binary consisting of distinct and disconnected forms is, fundamentally, a fallacy.

## Supplementary Material

XML Treatment for Solanum plastisexum
